# Associations between hedonic hunger and BMI during a two-year behavioural weight loss trial

**DOI:** 10.1371/journal.pone.0252110

**Published:** 2021-06-09

**Authors:** Bethan R. Mead, Emma J. Boyland, Paul Christiansen, Jason C. G. Halford, Susan A. Jebb, Amy L. Ahern

**Affiliations:** 1 Department of Psychology, University of Liverpool, Liverpool, United Kingdom; 2 Nuffield Department of Primary Care Health Sciences, University of Oxford, Oxford, United Kingdom; 3 MRC Epidemiology Unit, University of Cambridge, Cambridge, United Kingdom; King’s College London, UNITED KINGDOM

## Abstract

**Objective:**

Prospective studies on relationships between hedonic hunger and BMI (Body Mass Index) during weight management are lacking. This study examined if hedonic hunger reduced during a behavioural weight management programme, and if hedonic hunger predicted future BMI.

**Methods:**

Participants were 594 community-dwelling, UK-based adults(396 female; age 56.43 years, *s*.*d*. *=* 12.50, range 20–83 years); 490 participants (82.5%) had obesity. Participants were randomised to a 12- or 52-week behavioural weight management intervention (WW12 or WW52, respectively) or a brief self-help intervention (BI). Relationships between hedonic hunger and BMI over 24 months (baseline, 3, 12, 24 months) were analysed using an autoregressive cross-lagged model.

**Results:**

Hedonic hunger scores decreased from 2.71 (*s*.*d*. = .91) at baseline to 2.41 (*s*.*d*. = .88) at 3 months (*p* < .001, CI .22 to .38), remained reduced to 24 months, and were not affected by intervention arm at any time point (*p’*s>.05). Baseline hedonic hunger scores predicted 3-month scores (*B =* .76, *SE =* .03, *p* < .001, CI .71 to .82), 3-month scores predicted 12-month scores (*B* = .76, *SE* = .03, *p* < .001 CI .72 to .80), and 12-month scores predicted 24-month scores (*B* = .72, *SE* = .03, *p* < .001, CI .64 to .77). Higher hedonic hunger at 3 months predicted higher BMI at 12 months (*B* = .04, *SE* = .02, *p* = .03, CI .01 to .07) but not at 24 months (*p*>.05). BMI at 12 months was lower in WW52 30.87kg/m^2^, *s*.*d*. *=* 5.02) than WW12 (32.12 kg/m^2^, *s*.*d*. = 5.58, *p* = .02, CI .16 to 2.34) and BI (32.74 kg/m^2^, *s*.*d*. *=* 4.15, *p* = .01, CI .30 to 3.45). BMI was not affected by intervention at any other time point (*p*’s>.05).

**Conclusion:**

Hedonic hunger reduced during weight management irrespective of intervention. Early reductions in hedonic hunger appear to be associated with lower BMI in the medium-term. Identifying ways to reduce hedonic hunger during weight loss could aid weight management for some people.

## Introduction

The rising prevalence of obesity and consequences for health [1] have fuelled demand for efficacious, cost-effective and scalable weight management options [2]. Evidence suggests that at a group level, behavioural weight management programmes lead to moderate weight loss [3]. However, there is considerable, unexplained variability in treatment response such that these treatments appear to be effective for some people [4]. Identification of barriers may aid the development of tailored, successful weight management interventions.

The current food environment is a key driver of weight gain at a population level [5], but there are individual differences in the extent to which people respond to those cues. Specifically, the abundance of palatable, energy-dense foods that is characteristic of an obesogenic environment [6] has been suggested as a trigger for hedonic hunger [7]. Hedonic hunger refers to a motivation for palatable food in the absence of an energy deficit, driven by the pleasurable aspects of food, not homeostatic hunger [7]. Individual differences in hedonic hunger and the susceptibility to food cues may affect likelihood of achieving weight loss goals, but prospective studies that explore this are lacking.

Associations have been found between hedonic hunger, as measured by the Power of Food Scale (PFS) [8, 9], and appetite-related constructs that may present difficulties for individuals attempting to reduce weight. When sated, adults with obesity and high hedonic hunger who were exposed to food cues experienced greater craving for desired foods and reported less confidence in their ability to control their eating than those with lower hedonic hunger [10]. Neuroimaging data suggested that those with higher hedonic hunger may be predisposed to automatically process food cues and experience thoughts dominated by such cues, which may represent an additional challenge for people following a weight-management programme and in a food-deprived state [10]. In other studies, participants with elevated levels of both hedonic hunger and delay discounting (a measure of inhibitory control) showed greater food intake in a laboratory setting [11, 12]. In studies of adolescents [13] and adults [14, 15], elevated hedonic hunger appears to be associated with more frequent snacking, although greater self-regulatory abilities may reduce this effect [13, 15]. Higher hedonic hunger has also been associated with a greater likelihood of adolescents consuming fatty foods [16]. Elevated hedonic hunger was associated with greater eating in the absence of hunger (EAH) in lean women in a laboratory setting, suggesting that motivation for palatable food may trigger eating beyond satiety when such individuals are exposed to a palatable food [17]. Furthermore, higher hedonic hunger appears to predict the onset of loss of control eating (LOC) over two years in young women at risk of weight gain [18]. Associations between hedonic hunger and greater snacking, food intake and LOC may imply that having higher hedonic hunger could put an individual a greater risk of weight gain, but how this impacts efforts to reduce weight is understudied. Taken together, this body of evidence may suggest that heightened hedonic hunger impedes weight loss success because people wanting to lose weight would have to overcome their heightened drive for palatable food in order to adhere to eating patterns conducive to weight loss.

Individual differences in hedonic hunger may impact on the success of weight loss interventions, although the nature of this relationship is unclear. Post-operative bariatric surgery patients report lower levels of hedonic hunger than pre-operative patients with obesity and lean controls, however the nature of the relationship between hedonic hunger and weight loss over time and within participants was not explored in depth in these studies [19, 20]. Reductions in hedonic hunger following weight loss have also been seen in participants undergoing a behavioural weight management programme. O’Neil et al. [21] provide evidence for this in a trial of 111 participants referred to a commercial behavioural weight management programme (WW, formally Weight Watchers) for 12 weeks. During the intervention, hedonic hunger was reduced and greater reductions in hedonic hunger were associated with greater weight loss, particularly for participants with high hedonic hunger at baseline. Likewise, Theim et al. [22] reported greater reductions in hedonic hunger and weight loss in participants with high baseline hedonic hunger during a 15-week partial meal replacement trial. Taken together these findings suggest that reducing hedonic hunger in those most susceptible to it may improve weight loss outcomes, although the lack of a control intervention in these studies means the reductions cannot be definitively attributed to the intervention used. The current study expands on this by assessing weight loss and hedonic hunger during weight management interventions commonly used in healthcare settings [3] over 2 years. The sample we tested is larger and the length of follow up longer than those reported in existing studies of hedonic hunger and weight management [16, 21, 22], allowing for more extensive investigation of hedonic hunger and BMI (body mass index) than in previous literature.

Previous studies have typically measured hedonic hunger at daily and weekly intervals, or before and after short interventions (for example, 20 days [16] and 12- or 15 weeks [21, 22]). This means there is limited understanding of the relative stability of hedonic hunger or the relationship between changes in hedonic hunger and BMI over the medium-to long term. To date, only one study has considered the dynamics of the relationship between hedonic hunger and BMI during a longer weight management period. Cushing et al [23] reported that fluctuations in hedonic hunger mirror changes in BMI over a two year postoperative period in adolescents who have received bariatric surgery. However, the small sample (*N* = 16) and use of surgical weight loss in this study limits how generalisable these results may be to wider groups. Furthermore, the studies described above do not explicitly explore the temporal relationship between hedonic hunger and BMI over time, or the relevance of this to future weight.

The current study used the rigour of a randomised controlled trial (RCT) to explore the relationship between hedonic hunger and BMI during a behavioural weight management programme commonly used in healthcare settings [3]. Community-dwelling participants were randomised to the interventions for up to 1 year with follow up over 2 years. We investigated the effects of three weight management interventions (referral to commercial weight management for 12 or 52 weeks, or brief self-help intervention) on hedonic hunger. We used an autoregressive cross lagged model to examine the stability of hedonic hunger over time and to explore the temporal relationships between hedonic hunger and BMI, namely if and how hedonic hunger is associated with future BMI in the context of weight management trial. We hypothesised that reductions in hedonic hunger would be greater in participants undergoing behavioural weight management than a brief, self-help intervention. We also predicted that higher baseline hedonic hunger would be associated with higher future BMI during a weight management programme (indicating less successful weight loss). Finally, we predicted that hedonic hunger scores and BMI would decrease during the weight management trial. Increasing understanding of relationships between hedonic hunger and BMI during weight management has implications for intervention development; if hedonic hunger impedes weight management success, identifying and providing additional support to individuals with greater susceptibility to hedonic hunger may improve weight loss outcomes.

## Methods

### Measures and procedure

Data analysed in the current study were collected as part of the WRAP (Weight Loss Referrals for Adults in Primary Care) trial [ISRCTN82857232], which investigated the clinical and cost effectiveness of referral to a commercial weight management provider compared to standard care. The protocol, inclusion and exclusion criteria, and primary outcomes of the WRAP trial are described elsewhere [24, 25] so are presented here only in brief. Participants (*N* = 1,267) were recruited from GP (General Practitioner) practices in England and randomly allocated to one of the three intervention arms: brief self-help intervention (BI), referral to WW (formerly Weight Watchers) for 12 weeks (WW12), referral to WW for 52 weeks (WW52), in a 2:5:5 allocation ratio, stratified by gender and study centre (Liverpool, Oxford, Cambridge). Participants were eligible for inclusion if they were willing and able to fulfil study requirements and had a Body Mass Index (BMI) ≥28 kg/m^2^. Participants assigned to WW interventions were provided with vouchers to attend community-based WW group meetings and access to WW digital tools. Meetings include a confidential weigh in and a 30-minute interactive education session led by the coach that includes advice on diet, physical activity, positive mindset, using behavioural strategies (e.g. goal setting, self-monitoring, problem solving, modifying the personal food environment, and relapse prevention). The WW plan assigns numerical “points” values to foods and encourages participants to consume within a specified point limit each day. The BI was a booklet about healthy eating that was provided to the participant and explained by a researcher using a script [see 25, 26].

Participants attended trial visits at the research centre or their local GP practice at baseline (0 months), 3, 12 and 24 months where they completed clinical measurements including height (baseline only) and weight, from which BMI was calculated. Informed consent was obtained at the baseline visit. At or shortly before each visit participants completed the PFS [9]. The PFS is a validated fifteen item self-report measure assessing the effects of the food environment on motivation to consume palatable food. Overall scores on the PFS range from 1–5, with higher PFS total scores indicating higher hedonic hunger. This study used data from participants in the WRAP trial (*N* = 1,267) who provided complete PFS and weight data at every trial visit, resulting in a final sample of *n* = 594. We adopted a conservative, complete-case approach rather than imputing missing data. This is consistent with recent, similar work [15] and debate surrounding missing data and psychometric scales [26].

### Ethical approval

Ethical approval was received from NRES Committees for East of England Cambridge East (12/EE/0363), North West Liverpool Central (12/NW/0678), and South Central–Oxford (12/SC/0508).

### Analysis

Data analyses were performed using IBM SPSS 22.0 for Windows [27] and AMOS 22.0 [28].

A Pearson correlation was used to test for an association between baseline hedonic hunger and baseline BMI.

Mixed analysis of variance (ANOVA) was used to explore how hedonic hunger and BMI changed during and following the intervention, and if the intervention had a significant effect on such changes. Data were entered in to separate ANOVAs for BMI and hedonic hunger with time (4: baseline, 3 months, 12 months, 24 months) as the within subject factor and intervention group (3: WW12, WW52, BI) as the between subject factor. Significant effects (*p* < .05) were explored with Bonferroni corrected post hoc tests. Data for BMI and PFS total score violated the assumption of sphericity so the Greenhouse-Geisser correction was applied to degrees of freedom.

An autoregressive cross lagged model was used to examine the relationships between hedonic hunger (PFS total score) and BMI over time at each trial visit. Age at baseline and gender were included in the model as covariates. The fit of the proposed model was tested using multiple model fit indices. The standardised root mean residual (SRMR) absolute fit index was calculated wherein values < .08 were considered good [29]. Two non-centrality based indices were also computed- the Comparative fit index (CFI) where values ≥.95 were considered a good fit [29] and the root mean square error of approximation (RMSEA) where values ≤.08 [30] were considered a good fit. Finally two relative fit indices were reported: the incremental fit index (IFI) good fit >.90 [31]; normed fit index (NFI) good fit >.95 [32]. Exploratory data analysis of effects found in the model was carried out using bias corrected bootstrapping.

## Results

### Participant characteristics

Participants were 594 (396 female) adults and predominantly White-British (87.50%). Regarding employment status and highest level of education attained, the most frequently reported responses were “employed by other” (44.11%) and “GCSE level or equivalent” (completion of compulsory secondary education; 25.25%). Baseline participant characteristics are presented in Table 1 (see S1 Table for BMI and PFS scores split by time and intervention, and S2 Table for additional participant demographic characteristics). A series of one-way ANOVAs (analysis of variance) indicated that participants in each intervention arm were well-matched for baseline characteristics (age, BMI) and PFS total score (all *p*’s>.1).

**Table 1:** Mean (± standard deviation) and ranges of baseline age, BMI and PFS total score for all participants and WW12, WW52 and BI interventions.

		*Characteristic*					
*Intervention arm*	*N*	*Age at baseline*		*Baseline BMI*		*Baseline PFS total score*	
		Mean (s.d.)	*Range*	Mean (s.d.)	*Range*	Mean (s.d.)	*Range*
All	594	56.43 (±12.50)	20–83	34.04 (±4.85)	28.00–68.07	2.71 (±.91)	1.00–5.00
WW12	248	57.01 (±12.22)	24–81	34.14 (±5.03)	28.06–68.07	2.67 (±.87)	1.07–4.73
WW52	266	56.26 (±12.43)	26–83	33.92 (±4.91)	28.00–58.80	2.70 (±.94)	1.00–5.00
BI	80	55.21 (±13.23)	20–81	34.15 (±4.05)	28.65–49.52	2.83 (±.93)	1.13–4.80

Note: WW12 = referral to WW for 12 weeks; WW52 = referral to WW for 52 weeks; BI = brief self-help intervention.

### Sensitivity analysis

Due to blood sampling protocols as part of the main trial, some participants completed the PFS in a fasted state, which is not consistent with initial recommended conditions for hedonic hunger measurement [7]. The impact of fasting status on PFS scores was examined by comparing baseline PFS scores from participants at the Liverpool site who were fasted (*n* = 111; *Mdn* = 2.67, *s*.*d*. = .93) versus non-fasted (n = 24; *Mdn* = 2.64, *s*.*d*. = .95) when the measure was completed. No significant differences in scores (*U* = 1276.00, *z* = -.33, *p* = .75, *r* = .03) were found, so fasting status was not included as a covariate in subsequent analyses.

### Impact of intervention on changes in hedonic hunger and BMI over time

**Fig 1:**
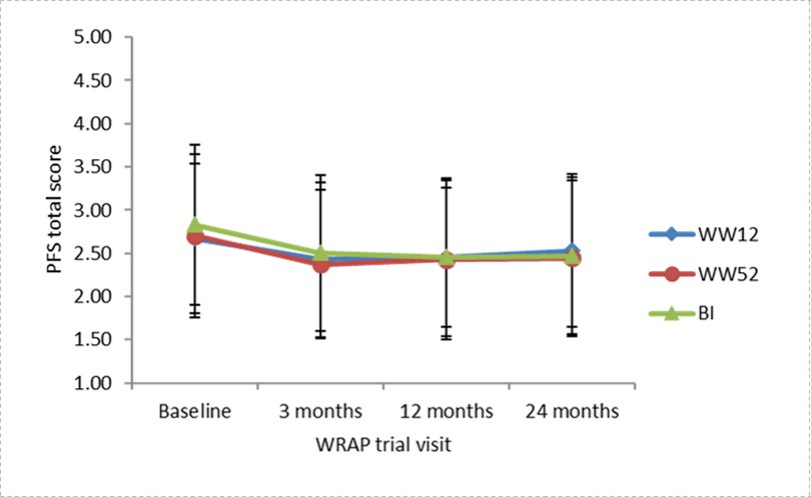
Mean PFS total score at each time point of the WRAP trial for each intervention. Error bars represent ±1 standard deviation.

There was a main effect of time (baseline, 3, 12, 24 months) on hedonic hunger (*F* (2.961) = 42.55, *p* < .001, η_p_^2^ = .07), but no significant effect of intervention arm on hedonic hunger or a significant intervention arm x time interaction (*p*’s>.05). Bonferroni post-hoc tests revealed that the main effect of time was driven by mean PFS total scores decreasing from 2.71 (*s*.*d*. = .91) at baseline to 2.41 (*s*.*d*. = .88) at 3 months (*p* < .001, CI .22 to .38). Fig 1 displays mean PFS total scores for each intervention arm. PFS total scores decreased at 3 months and remained significantly lower than baseline at 12 months (2.44, *s*.*d*. = .90, *p* < .001, CI .21 to .38) and 24 months (2.48, *s*.*d*. = .89; *p* < .001, CI .17 to .34).

**Fig 2:**
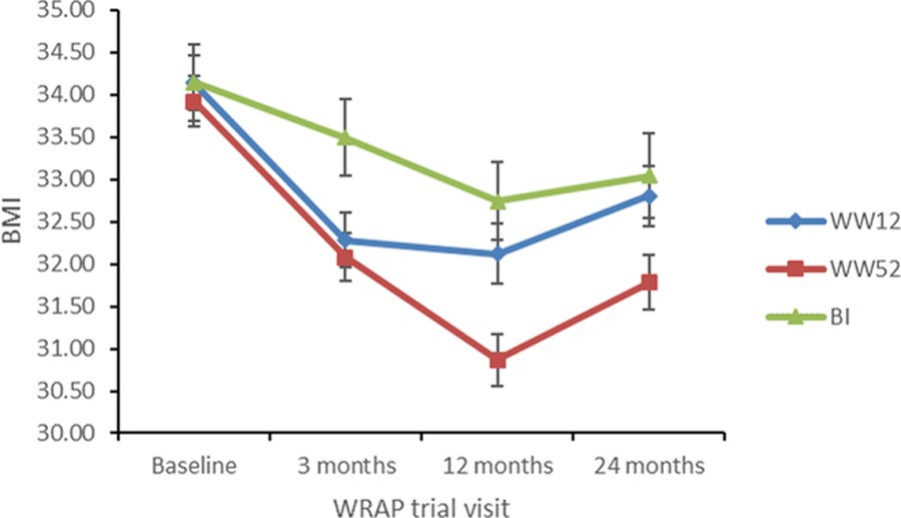
Mean BMI at each time point of the WRAP trial for each intervention. Error bars represent ±1 standard deviation.

There was a significant main effect of time (baseline, 3, 12, 24 months) on BMI (*F* (1.67) = 128.71, *p* < .001, η_p_^2^ = .18) and a significant intervention arm x time interaction (*F* (3.33) = 10.26, *p* < .001, η_p_^2^ = .03). There was no significant main effect of intervention arm on BMI (*p*>.05). Bonferroni corrected post-hoc tests revealed that at 12 months mean BMI for participants in the 52-week programme (30.87kg/m^2^, *s*.*d*. *=* 5.02) was significantly lower than the 12-week programme (32.12 kg/m^2^, *s*.*d*. = 5.58, *p* = .02, CI .16 to 2.34) and BI (32.74 kg/m^2^, *s*.*d*. *=* 4.15, *p* = .01, CI .30 to 3.45). There was no significant difference in 12-month BMI between the 12-week programme and BI (*p*>.05). There were no significant differences in BMI between interventions at 24 months (all *p*’s >.05). Fig 2 displays mean BMI for each intervention arm.

### Relationships between hedonic hunger and BMI over time

There was a small positive correlation between baseline PFS score and BMI for the whole sample (*r* = .11, *p* < .01), indicating that a higher baseline PFS total score was associated with a higher baseline BMI.

As there was no effect of intervention arm on hedonic hunger and no interaction between intervention arm and time, data were collapsed across interventions to examine the relationship between hedonic hunger and BMI over time using an autoregressive cross lagged model. Model fit indices demonstrated that the tested model was a good fit to the data (CFI = .95; IFI = .95; NFI = .95; SRMR = .048) apart from the RMSEA that was beyond the acceptable range (RMSEA = .19). The discrepancy between the CFI and RMSEA non-centrality based indices was the result of the model fitting the necessary conditions for RMSEA>.1 and CFI >.95, as outlined by Lai and Green [33]. This indicates that the high RMSEA value is due to the product of the fit functions calculations rather than poor model fit, which is also supported by the good fit of the relative, and absolute fit, indices. Post-hoc power analysis for the autoregressive model indicates that the analysis had an acceptable level of power (80%, *p* < .05) with *N* = 579 as the minimum sample size required for this analysis [34].

**Fig 3:**
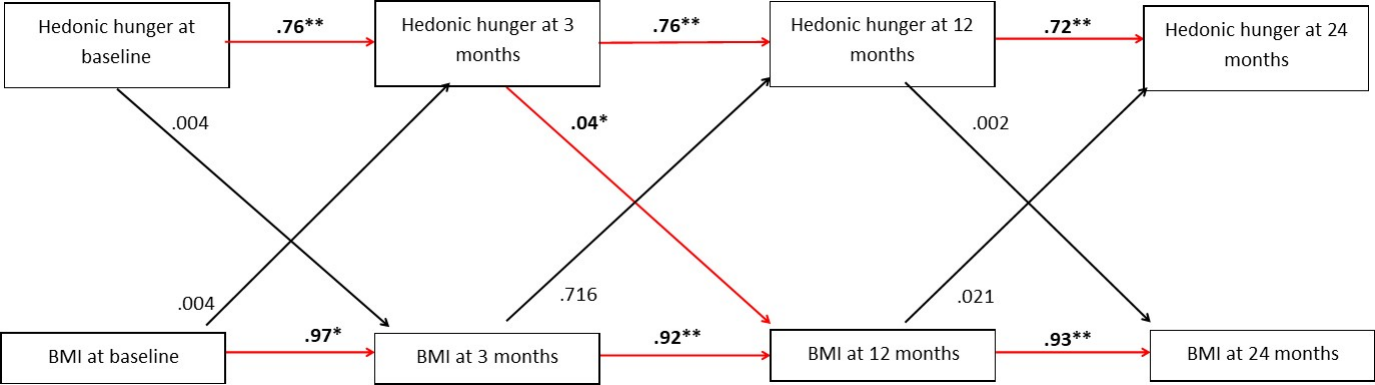
Model of the relationships between hedonic hunger and BMI at baseline, 3, 12 and 24 months (standardized regression coefficients reported). Covariates (age at baseline and gender) and error terms not shown for clarity. Significant pathways are marked in red. Pathways marked * were significant at *p <* .05; those marked ** were significant at *p* < .001. Pathways marked in black were non-significant.

PFS total scores at each time point predicted scores at the subsequent time point (shown in Fig 3). Baseline PFS total scores predicted 3-month scores (*B =* .76, *SE =* .03, *p* < .001, CI = .71 to .82), 3-month scores predicted 12-month scores (*B* = .76, *SE* = .03, *p* < .001 CI = .72 to .80), and 12-month scores predicted 24-month scores (*B* = .72, *SE* = .03, *p* < .001, CI = .64 to .77). BMI at each visit was also predictive of BMI at the subsequent visit; baseline BMI predicted 3-month BMI (*B* = .97, *SE* = .01, *p* < .001, CI = .95 to .99), 3-month BMI predicted 12-month BMI (*B* = .92, *SE* = .02, *p* < .001, CI = .90 to .96), and 12-month BMI predicted 24-month BMI (*B =* .93, *SE* = .01, *p* < .001, CI .90 to .95).

Baseline PFS total score did not predict BMI at 3, 12 or 24 months. The direct pathway between baseline PFS total score and 3-month BMI was nonsignificant (*B* = .004, *SE* = .01, *p* = .89, CI = -.02 to .02). Bias corrected bootstrap confidence intervals were calculated, and no significant indirect pathways between baseline PFS and 12-month BMI (*B* = .03, *SE* = .02, *p* = .13, CI = -.002 to .063) or 24-month BMI (*B* = .03, *SE* = .019, *p* = .10, CI = -.00 to .06) were evident.

Cross-lagged pathways between hedonic hunger and BMI at baseline, 3, 12 and 24 months were tested to investigate changes in relationships over time. One significant cross-lagged pathway emerged: PFS total score at 3 months predicted BMI at 12 months (*B* = .04, *SE* = .02, *p* = .030, CI = .01 to .07). No other cross-lagged pathways were significant (*p*’s>.05). Therefore, after controlling for variance associated with BMI at 3 months, a higher hedonic hunger score at 3 months was predictive of a higher BMI at 12 months, and there is no evidence for significant pathways between hedonic hunger and BMI at other time points. Additional details concerning the effects of covariates (baseline age and gender) are presented in S2 Table. We also conducted a supplementary analysis of the model presented in Fig 3. This analysis investigated the relationships between PFS scores and weight (kg), as opposed to BMI. The overall pattern of result from this analysis is consistent with the findings presented here. Details of this analysis are shown in S1 Appendix.

## Discussion

During this 2-year trial of three weight management interventions, hedonic hunger and BMI reduced between baseline and 3 months, and both remained suppressed to 24 months. Hedonic hunger reduced in all groups and there was no intervention-specific effect. While baseline hedonic hunger did not predict BMI at any time point, higher hedonic hunger score at 3 months predicted a higher BMI at 12 months. This pattern of results remained consisted when analyses were repeated with weight, as opposed to BMI. This suggests that early changes in hedonic hunger during the first 12 weeks of a weight loss programme are associated with BMI in the medium term (12 months), but not beyond this (24 months).

The reduction in hedonic hunger during weight management is consistent with previous findings [20–23], including the results from a 12-week behavioural weight management intervention [21]. However, whereas a lack of control group [21] has precluded attributions of these effects to a specific intervention, the current study shows the reduction in PFS scores is common across all intervention arms. This implies it is weight loss itself (or attempts to lose weight), rather than referral to a behavioural weight management programme per se, that may reduce hedonic hunger. Although the magnitude of the decrease in PFS scores we report here is small (-0.30 on a scale with a possible score range of 1 to 5), others have reported comparable reductions of -0.46 [21] and -0.57 [22] during interventions of similar length. A possible explanation for this effect may be that the behavioural and cognitive changes people on a weight loss programme may undergo during weight loss attempts, such as identifying and managing sources of temptation and strengthening inhibitory self-control skills to manage this, may reduce hedonic hunger. These could result in reduced intake of energy dense food, aiding weight loss. Indeed, others have recently highlights the interplay between hedonic hunger, self-control and overeating [15], although these hypotheses need to be tested further.

There was a small, positive association between baseline PFS scores and BMI, indicating that participants with higher hedonic hunger at the start of the trial also had a higher BMI. This small correlation is consistent with research that indicates a weak PFS-BMI relationship [9, 35, 36], although others have not found such a correlation [11, 37, 38] or suggest hedonic hunger is more clearly associated with weight classification than BMI [35, 39]. This may reflect heterogeneity in study samples, or it may indicate that higher hedonic hunger is not unique to elevated BMIs, and other factors (such as self-regulatory abilities) interact with hedonic hunger to influence appetite and body weight. For example, others have shown that such hedonic hunger and self-regulatory abilities interact to influence laboratory food intake, so it is plausible that these effects translate to behaviour that promotes increased BMI [11, 12].

The 24-month study period is longer than most existing research on hedonic hunger within the context of weight management interventions [15, 19–21, although see 23] and is a strength of this study. Our findings indicate that hedonic hunger as a construct is relatively stable over a two-year period, and the PFS at each time point predicted PFS scores at the subsequent time point. This was demonstrated even though overall PFS score decreased with during the weight management trial, suggesting hedonic hunger is a modifiable factor and supporting the idea that this construct is more trait- than state-like. Early changes in hedonic hunger appear to associated with future BMI in the medium term, so future work may wish to explore if behavioural interventions that target reductions in hedonic hunger improve weight loss in those who report elevated hedonic hunger.

Taken together with the existing literature, findings suggest that the role of hedonic hunger in weight control may be moderated by self-regulatory processes. Dual-process models of obesity [40–42] posit that obesity may arise from an imbalance in impulsive and reflective processes. Hedonic hunger reflects the drive for food for pleasure and could be viewed as similar to an impulsive process, that results from repeated associations between cues (e.g. food) and rewarding outcomes (e.g. pleasure from eating). Others have suggested that those with elevated hedonic hunger may be prone to automatically process food cues [10], which could be driven by heightened sensitivity to their potentially rewarding properties [43] and interpreted as an indicator of an impulsive process. Conversely, self-regulatory skills associated with successful dieting are reflective processes that work to inhibit impulsive responses [42]. Findings show hedonic hunger interacts with self-regulatory measures to influence appetite, with greater hedonic hunger and less effective self-regulation proposed to represent vulnerability for increased appetite [11–13, 15]. Future research should seek to identify methods for specifically targeting reductions in hedonic hunger to inform tailored weight management interventions for people who are susceptible to these impulsive processes. This may be through directly reducing susceptibility to hedonic hunger or enhancing self-regulatory skills, though this was not assessed here.

There was no relationship between hedonic hunger at any time point and BMI at 24 months. Between 12- and 24-months participants in the WRAP trial were not receiving any intervention. An explanation for this may be that hedonic hunger’s influence on BMI is most pronounced during early stages of a weight management programme, perhaps when weight management goals are being established. Between 12- and 24-months other factors may have become more salient to weight management. Future research focussed specifically on weight maintenance may wish to measure hedonic hunger alongside other characteristics or behaviours, such as food choice and temptation management, to understand this further.

### Limitations

Complete PFS and BMI data were only available for 594 of the 1,267 participants randomised in the WRAP trial. This was due to participants not completing questionnaires and attrition, although overall WRAP trial retention rates were good [25]. This is expected during long-term trials, but this may limit the strength of our findings. We acknowledge that, due to the large amount of missing data, we are unable to explore if additional factors or participant characteristics related to the likelihood of completing the WRAP trial may influence these results. Future work is needed to confirm these findings in larger or more complete samples.

A further limitation is the homogeneity of our sample. Most participants described themselves as White-British, and all were recruited in England. As a result, we are unable to speculate if intercultural differences may influence our findings. More research with more diverse population samples is needed to understand this.

Finally, two studies have provided suggested population norms of 1.94 [44] and 2.07 [36] for the PFS total score. The PFS total scores we report at each measurement point in this study are notably higher than these. This may imply that our sample reported, on average, elevated hedonic hunger at each time point and limit the generalisability of our findings. However, these norms are taken from population samples, not treatment-seeking participants, and may not be reflective of the experience of hedonic hunger in individuals embarking on weight management attempts. PFS total scores in our study were more comparable to those reported during a similar intervention [22], so we tentatively suggest that the levels of hedonic hunger experienced by participants in this study were akin to other treatment-seeking individuals with overweight or obesity during a weight management trial. As no norms for such samples have been established it would be helpful for future research to develop these.

## Conclusion

These data show that hedonic hunger reduces during weight management programmes; this does not seem to be attributable to a specific effect of any of the interventions studied here. While average reductions in hedonic hunger are small, early changes in hedonic hunger appear to be associated with future BMI in the medium term. Results tentatively suggest that for some people greater support to reduce hedonic hunger during weight loss may enable them to better navigate the obesogenic food environment and thus aid successful weight management.

## Supporting information

S1 Table Mean (± standard deviation) mean baseline BMI and PFS total score at each time point for WW12, WW52 and BI interventions. (DOCX)

S2 Table Additional participant demographic characteristics. (DOCX)

S1 Appendix Additional analyses. (DOCX)

## References

[1] Wang H, Naghavi M, Allen C, Barber RM, Bhutta ZA, Carter A, et al. Global, regional, and national life expectancy, all-cause mortality, and cause-specific mortality for 249 causes of death, 1980–2015: a systematic analysis for the Global Burden of Disease Study 2015. Lancet. 2016 Oct;388(10053):1459–544. doi: 10.1016/S0140-6736(16)31012-1 27733281 PMC5388903

[2] Bray GA, Kim KK, Wilding JPH. Obesity: a chronic relapsing progressive disease process. A position statement of the World Obesity Federation. Obes Rev. 2017 May 10; doi: 10.1111/obr.12551 28489290

[3] Hartmann-Boyce J, Johns DJ, Jebb SA, Summerbell C, Aveyard P, Behavioural Weight Management Review Group. Behavioural weight management programmes for adults assessed by trials conducted in everyday contexts: systematic review and meta-analysis. Obes Rev. 2014 Nov 1;15(11):920–32. doi: 10.1111/obr.12220 25112559 PMC4233997

[4] Teixeira PJ, Carraça E V, Marques MM, Rutter H, Oppert J-M, De Bourdeaudhuij I, et al. Successful behavior change in obesity interventions in adults: a systematic review of self-regulation mediators. BMC Med. 2015 Dec 16;13(1):84. doi: 10.1186/s12916-015-0323-6 25907778 PMC4408562

[5] Swinburn BA, Sacks G, Hall KD, McPherson K, Finegood DT, Moodie ML, et al. The global obesity pandemic: shaped by global drivers and local environments. Lancet. 2011;378(9793):804–14. doi: 10.1016/S0140-6736(11)60813-1 21872749

[6] Swinburn B, Egger G, Raza F. Dissecting obesogenic environments: The development and application of a framework for identifying and prioritizing environmental interventions for obesity. Prev Med (Baltim). 1999 Dec;29(6 I):563–70. doi: 10.1006/pmed.1999.0585 10600438

[7] Lowe MR, Butryn ML. Hedonic hunger: a new dimension of appetite? Physiol Behav. 2007 Jul 24;91(4):432–9. doi: 10.1016/j.physbeh.2007.04.006 17531274

[8] Lowe MR, Butryn ML, Didie ER, Annunziato RA, Thomas JG, Crerand CE, et al. The Power of Food Scale. A new measure of the psychological influence of the food environment. Appetite. 2009 Aug;53(1):114–8. doi: 10.1016/j.appet.2009.05.016 19500623

[9] Cappelleri JC, Bushmakin a G, Gerber R a, Leidy NK, Sexton CC, Karlsson J, et al. Evaluating the Power of Food Scale in obese subjects and a general sample of individuals: development and measurement properties. Int J Obes (Lond). 2009 Aug;33(8):913–22. doi: 10.1038/ijo.2009.107 19506564

[10] Rejeski WJ, Burdette J, Burns M, Morgan AR, Hayasaka S, Norris J, et al. Power of food moderates food craving, perceived control, and brain networks following a short-term post-absorptive state in older adults. Appetite. 2012 Jun;58(3):806–13. doi: 10.1016/j.appet.2012.01.025 22329987 PMC3340490

[11] Appelhans BM, Woolf K, Pagoto SL, Schneider KL, Whited MC, Liebman R. Inhibiting food reward: delay discounting, food reward sensitivity, and palatable food intake in overweight and obese women. Obesity (Silver Spring). 2011 Nov;19(11):2175–82. 21475139 10.1038/oby.2011.57 PMC3303186

[12] Ely A V, Howard J, Lowe MR. Delayed discounting and hedonic hunger in the prediction of lab-based eating behavior. Eat Behav. 2015 Dec;19:72–5. doi: 10.1016/j.eatbeh.2015.06.015 26183899

[13] Stok FM, De Vet E, Wardle J, Chu MT, De Wit J, De Ridder DTD. Navigating the obesogenic environment: How psychological sensitivity to the food environment and self-regulatory competence are associated with adolescent unhealthy snacking. Eat Behav. 2014 Dec 10;17C:19–22. doi: 10.1016/j.eatbeh.2014.12.003 25528719

[14] Schüz B, Schüz N, Ferguson SG. It’s the power of food: individual differences in food cue responsiveness and snacking in everyday life. Int J Behav Nutr Phys Act. 2015 Jan 7;12(1):149. 26643690 10.1186/s12966-015-0312-3 PMC4672526

[15] Horwath CC, Hagmann D, Hartmann C. The Power of Food: Self-control moderates the association of hedonic hunger with overeating, snacking frequency and palatable food intake. Eat Behav. 2020 May;38:101393. doi: 10.1016/j.eatbeh.2020.101393 32497904

[16] Bejarano CM, Cushing CC. Dietary Motivation and Hedonic Hunger Predict Palatable Food Consumption: An Intensive Longitudinal Study of Adolescents. Ann Behav Med. 2018 Aug 16;52(9):773–86. doi: 10.1093/abm/kax051 30124763

[17] Feig EH, Piers AD, Kral TVE, Lowe MR. Eating in the absence of hunger is related to loss-of-control eating, hedonic hunger, and short-term weight gain in normal-weight women. Appetite. 2018 Apr 1;123:317–24. doi: 10.1016/j.appet.2018.01.013 29331366

[18] Lowe MR, Arigo D, Butryn ML, Gilbert JR, Sarwer D, Stice E. Hedonic Hunger Prospectively Predicts Onset and Maintenance of Loss of Control Eating Among College Women. Heal Psychol. 2016 Dec 21;35(3):238–44. doi: 10.1037/hea0000291 26690638 PMC4859341

[19] Ullrich J, Ernst B, Wilms B, Thurnheer M, Hallschmid M, Schultes B. The Hedonic Drive to Consume Palatable Foods Appears to be Lower in Gastric Band Carriers than in Severely Obese Patients Who Have Not Undergone a Bariatric Surgery. Obes Surg. 2013 Apr;23(4):474–9. doi: 10.1007/s11695-012-0818-6 23179243

[20] Ullrich J, Ernst B, Wilms B, Thurnheer M, Schultes B. Roux-en Y gastric bypass surgery reduces hedonic hunger and improves dietary habits in severely obese subjects. Obes Surg. 2013 Jan;23(1):50–5. doi: 10.1007/s11695-012-0754-5 22941334

[21] O’Neil PM, Theim KR, Boeka A, Johnson G, Miller-Kovach K. Changes in weight control behaviors and hedonic hunger during a 12-week commercial weight loss program. Eat Behav. 2012 Dec;13(4):354–60. doi: 10.1016/j.eatbeh.2012.06.002 23121787

[22] Theim KR, Brown JD, Juarascio AS, Malcolm RR, O’Neil PM. Relations of hedonic hunger and behavioral change to weight loss among adults in a behavioral weight loss program utilizing meal-replacement products. Behav Modif. 2013 Nov;37(6):790–805. doi: 10.1177/0145445513501319 24013101

[23] Cushing CC, Benoit SC, Peugh JL, Reiter-Purtill J, Inge TH, Zeller MH. Longitudinal trends in hedonic hunger after Roux-en-Y gastric bypass in adolescents. Surg Obes Relat Dis. 2014;10(1):125–30. doi: 10.1016/j.soard.2013.05.009 24135561 PMC4420196

[24] Ahern AL, Aveyard PN, Halford JC, Mander A, Cresswell L, Cohn SR, et al. Weight loss referrals for adults in primary care (WRAP): protocol for a multi-centre randomised controlled trial comparing the clinical and cost-effectiveness of primary care referral to a commercial weight loss provider for 12 weeks, referral for 52 week. BMC Public Health. 2014 Jan;14:620. doi: 10.1186/1471-2458-14-620 24943673 PMC4230033

[25] Ahern AL, Wheeler GM, Aveyard P, Boyland EJ, Halford JCG, Mander AP, et al. Extended and standard duration weight-loss programme referrals for adults in primary care (WRAP): a randomised controlled trial. Lancet. 2017; doi: 10.1016/S0140-6736(17)30647-5 28478041 PMC5459752

[26] Robinson MA. Using multi-item psychometric scales for research and practice in human resource management. Hum Resour Manage. 2018 May 1;57(3):739–50.

[27] IBM Corp. IBM SPSS Statistics for Windows, Version 22.0. Armonk, NY; 2013.

[28] Arbuckle JL. IBM ® SPSS ® Amos TM 22 User’s Guide. 2013.

[29] Hu L, Bentler PM. Cutoff criteria for fit indexes in covariance structure analysis: Conventional criteria versus new alternatives. Struct Equ Model A Multidiscip J. 1999 Jan;6(1):1–55.

[30] Browne MW, Cudeck R. Alternative Ways of Assessing Model Fit. Sociol Methods Res. 1992 Nov;21(2):230–58.

[31] Bollen KA. A New Incremental Fit Index for General Structural Equation Models. Sociol Methods Res. 1989 Feb;17(3):303–16.

[32] Bentler PM, Bonett DG. Significance tests and goodness of fit in the analysis of covariance structures. Psychol Bull. 1980;88(3):588–606.

[33] Lai K, Green SB. The Problem with Having Two Watches: Assessment of Fit When RMSEA and CFI Disagree. Multivariate Behav Res. 2016 May 3;51(2–3):220–39. doi: 10.1080/00273171.2015.1134306 27014948

[34] Kim KH. The relation among fit indexes, power, and sample size in structural equation modeling. Vol. 12, Structural Equation Modeling. Lawrence Erlbaum Associates, Inc.; 2005. p. 368–90.

[35] Ribeiro G, Camacho M, Santos O, Pontes C, Torres S, Oliveira-Maia AJ. Association between hedonic hunger and body-mass index versus obesity status. Sci Rep. 2018 Dec 11;8(1):5857. doi: 10.1038/s41598-018-23988-x 29643337 PMC5895788

[36] Andreeva E, Neumann M, Nöhre M, Brähler E, Hilbert A, de Zwaan M. Validation of the German Version of the Power of Food Scale in a General Population Sample. Obes Facts. 2019 Jul 2;12(4):1–11. 31266028 10.1159/000500489 PMC6758710

[37] Witt A a, Raggio G a, Butryn ML, Lowe MR. Do hunger and exposure to food affect scores on a measure of hedonic hunger? An experimental study. Appetite. 2014 Mar;74:1–5. doi: 10.1016/j.appet.2013.11.010 24269255

[38] Yoshikawa T, Orita K, Watanabe Y, Tanaka M. Validation of the Japanese Version of the Power of Food Scale in a Young Adult Population. Psychol Rep. 2012 Aug 1;111(1):253–65. doi: 10.2466/08.02.06.15.PR0.111.4.253-265 23045867

[39] Thomas E a, Bechtell JL, Vestal BE, Johnson SL, Bessesen DH, Tregellas JR, et al. Eating-related behaviors and appetite during energy imbalance in obese-prone and obese-resistant individuals. Appetite. 2013 Jun;65:96–102. doi: 10.1016/j.appet.2013.01.015 23402714 PMC3622833

[40] Hofmann W, Friese M, Strack F. Impulse and Self-Control From a Dual-Systems Perspective. Perspect Psychol Sci. 2009 Mar 1;4(2):162–76. doi: 10.1111/j.1745-6924.2009.01116.x 26158943

[41] Strack F, Deutsch R. Reflective and Impulsive Determinants of Social Behavior. Personal Soc Psychol Rev. 2004 Aug 21;8(3):220–47. doi: 10.1207/s15327957pspr0803_1 15454347

[42] Jones A, Hardman CA, Lawrence N, Field M. Cognitive training as a potential treatment for overweight and obesity: A critical review of the evidence. Appetite. 2018 May 1;124:50–67. doi: 10.1016/j.appet.2017.05.032 28546010

[43] Espel-Huynh HM, Muratore AF, Lowe MR. A narrative review of the construct of hedonic hunger and its measurement by the Power of Food Scale. Obes Sci Pract. 2018 Jun;4(3):238–49. doi: 10.1002/osp4.161 29951214 PMC6009994

[44] Ribeiro G, Santos O, Camacho M, Torres S, Mucha-Vieira F, Sampaio D, et al. Translation, Cultural Adaptation and Validation of the Power of Food Scale for Use by Adult Populations in Portugal. Vol. 28, Acta Médica Portuguesa. 2015. p. 575–82. doi: 10.20344/amp.6517 26667860

