# Supplementary material for: Associations between hedonic hunger and BMI during a two-year behavioural weight loss trial
Source: PLoS One. 2021 Jun 9;16(6):e0252110. doi: 10.1371/journal.pone.0252110 (PMC8189467; doi:10.1371/journal.pone.0252110)
Supplement: S1 Table — (DOCX) [file pone.0252110.s001.docx]

S1 Table. *Mean (± standard deviation) mean baseline BMI and PFS total score at each time point for WW12, WW52 and BI interventions.*

|  | | *BMI* | | | | | | *PFS* | | | | | |  | |  | |  |  |
| --- | --- | --- | --- | --- | --- | --- | --- | --- | --- | --- | --- | --- | --- | --- | --- | --- | --- | --- | --- |
|  | | *Intervention group* | | | | | | *Intervention group* | | | | | |  | |  | |  |  |
| *WRAP trial visit* | | WW12 | | WW52 | | BI | | WW12 | | WW52 | | BI | |  | |  | |  |  |
| Baseline | | 34.14 (±5.03) | | 33.92 (±4.91 | | 34.15 (±4.06) | | 2.67 (±0.87) | | 2.70 (±0.94) | | 2.83 (±0.93) | |  | |  | |  |  |
| 3 months | | 32.29 (±5.15) | | 32.08 (±4.61) | | 33.49 (±4.03) | | 2.42 (±0.89) | | 2.37 (±0.86) | | 2.51 (±0.90) | |  | |  | |  |  |
| 12 months | | 32.12 (±5.58) | | 30.87 (±5.02) | | 32.74 (±4.15) | | 2.45 (±0.92) | | 2.43 (±0.92) | | 2.45 (±0.80) | |  | |  | |  |  |
| 24 months | | 32.81 (±5.56) | | 31.78 (±5.25) | | 33.04 (±4.50) | | 2.53 (±0.88) | | 2.44 (±0.90) | | 2.47 (±0.91) | |  | |  | |  |  |
|  | |  | |  | |  | |  | |  | |  | |  | |  | |  |  |
|  | |  | |  | |  | |  | |  | |  | |  | |  | |  |  |
|  |  | |  | |  | |  | |  | |  | |  | |  | |  |  |  |
|  |  | |  | |  | |  | |  | |  | |  | |  | |  |  |  |
