# Supplementary material for: Associations between hedonic hunger and BMI during a two-year behavioural weight loss trial
Source: PLoS One. 2021 Jun 9;16(6):e0252110. doi: 10.1371/journal.pone.0252110 (PMC8189467; doi:10.1371/journal.pone.0252110)
Supplement: S2 Table — (DOCX) [file pone.0252110.s002.docx]

S2 Table. *Additional participant demographic characteristics.*

| *Characteristic* | | *Frequency* | *%* |
| --- | --- | --- | --- |
| Employment type | Employed by other | 262 | 44.11 |
|  | Other | 20 | 3.37 |
|  | Prefer not to say | 1 | 0.17 |
|  | Retired | 225 | 37.88 |
|  | Self-Employed | 43 | 7.24 |
|  | Student | 4 | 0.67 |
|  | Unable to work | 19 | 3.2 |
|  | Unemployed | 17 | 2.86 |
| Highest level of education attained | None stated | 53 | 8.92 |
|  | None | 29 | 4.88 |
|  | GCSE or equivalent | 150 | 25.25 |
|  | A-Level or equivalent | 114 | 19.19 |
|  | Post-secondary study | 18 | 3.03 |
|  | University degree or equivalent | 128 | 21.55 |
|  | Higher degree or equivalent | 102 | 17.17 |
| Ethnicity | Asian or Asian-British - Bangladeshi | 2 | 0.34 |
|  | Asian or Asian-British - Indian | 7 | 1.18 |
|  | Asian or Asian-British - Pakistani | 1 | 0.17 |
|  | Black or Black-British - African | 4 | 0.67 |
|  | Black or Black-British - Caribbean | 3 | 0.51 |
|  | Black or Black-British - Other | 1 | 0.17 |
|  | Chinese | 2 | 0.34 |
|  | Mixed - Other | 2 | 0.34 |
|  | Mixed - White and Asian | 1 | 0.17 |
|  | Mixed - White and Black African | 1 | 0.17 |
|  | Mixed - White and Black Caribbean | 1 | 0.17 |
|  | Not stated | 1 | 0.17 |
|  | Other | 7 | 1.18 |
|  | Prefer not to say | 6 | 1.01 |
|  | White-British | 520 | 87.54 |
|  | White-Irish | 8 | 1.35 |
|  | White-Other | 27 | 4.55 |
