# Supplementary material for: Associations between hedonic hunger and BMI during a two-year behavioural weight loss trial
Source: PLoS One. 2021 Jun 9;16(6):e0252110. doi: 10.1371/journal.pone.0252110 (PMC8189467; doi:10.1371/journal.pone.0252110)
Supplement: S1 Appendix — (DOCX) [file pone.0252110.s003.docx]

**S1 Appendix**

**Effect of age and gender on PFS scores and BMI – additional analysis from the autoregressive cross lagged model.**

Age was a significant predictor of PFS total scores at 3 months (*B* = -.06, *SE* = .03, *p=*.035, *CI* = -.10 to -.02) and at 24 months (*B* = -.09, *SE* = .03, *p=*.003, *CI* = -.13 to -.04), and of BMI at 12 months (*B* = -.05, *SE* = .02, *p=*.005, *CI* = -.80 to -.02). Gender was a significant predictor of BMI at 12 months (*B* = .13, *SE* = .04, *p*<.001, *CI* = .03 to .09). Age and gender did not predict PFS scores or BMI at any other time points.

**Relationships between hedonic hunger and weight over time**

In response to reviewer queries, we conducted a supplementary analysis of the autoregressive cross-lagged model presented in Figure 3 to explore relationships between PFS total scores and weight (kilograms; kg; as opposed to BMI). Age at baseline (years) and gender were entered in to the model as covariates. Model fit indices are shown in S3 Table. This model was a good fit of the data and the overall pattern of results is consistent with the findings presented in the main manuscript, indicating that our findings are applicable to weight and BMI.

S3 Table. *Model fit indices for 1. autoregressive model using weight (kg; main manuscript) and 2. Primary analyses in the manuscript - autoregressive model using BMI data.*

| *Model fit index* | *1. Autoregressive model using weight (kg) data* | *2. Primary analyses in the manuscript (BMI)* |
| --- | --- | --- |
| *SRMR* | *.047* | *.048* |
| *CFI* | *.96* | *.95* |
| *IFI* | *.96* | *.95* |
| *NFI* | *.96* | *.95* |
| *RMSEA* | *.19* | *.19* |
| *AIC* | *374.524* | *382.38* |

*Note. SRMR= standardised root mean residual; CFI = comparative fit index; IFI = Incremental Fit Index; NFI = Normed fit index; RMSEA = Root mean square error of approximation.*

The overall pattern of results from models 1 and 2 are similar in direction. When weight data is entered in to the model in place of BMI, PFS total scores at each time point predicted scores at the subsequent time point. Baseline PFS total scores predicted 3-month scores (*B*=.76, *SE*=.03, *p*<.001, *CI* = .71 to .81), 3-month scores predicted 12-month scores (*B*=.76, *SE*=.03, *p*<.001, *CI* = .71 to .81), and 12-month scores predicted 24-month scores (*B*=.71, *SE*=.03, *p*<.001, *CI* = .65 to .78). Weight at each visit is also predictive of weight at the subsequent visit; baseline weight predicted 3-month weight (*B*=.97, *SE*=.01, *p*<.001, *CI* = .95 to .99), 3-month weight predicted 12-month weight (*B*=.90, *SE*=.02, *p*<.001, *CI* =.87 to .93), and 12-month weight predicted 24-month weight (*B*=.95, *SE*=.01, *p*<.001, *CI* = .92 to .97).

Baseline PFS total score did not predict weight at 3, 12 or 24 months (*p*’s>.05). The direct pathway between baseline PFS total score and 3-month weight was nonsignificant (*B*=.002, *SE* = .02, *p*=.83, *CI* = -.01 to .02). Bias corrected bootstrap confidence intervals were calculated, and no significant indirect pathways between baseline PFS and 12-month weight (*B*=.03, *SE* =.02, *p*=.13, *CI* =-.00 to .057) or 24-month weight (*B*=.03, *SE* = .02, *p*=.09, *CI* = .00 to .056) were evident.

Cross-lagged pathways between hedonic hunger and weight at baseline, 3, 12 and 24 months were tested to investigate changes in relationships over time. As shown in the model that used BMI data, only one significant cross-lagged pathway emerged: PFS total score at 3 months predicted weigh at 12 months (*B*=.03, *SE*=.02, *p*=.030, *CI* = .01 to .06). No other cross-lagged pathways were significant (*p*’s>.05).

Age was a marginally significant predictor of PFS total scores at 3 months (*B* = -.06, *SE* = .03, *p=*.051, *CI* = -.1 to -.01), and a significant predictor of PFS total score at 24 months (*B* = -.09, *SE* = .03, *p=*.005, CI = -.14 to -.04), and of weight at 12 months (*B* = -.05, *SE* = .02, *p=*.006, CI = -.08 to -.02). Gender was a significant predictor of weight at 12 months (*B* = .19, *SE* = .04, *p*<.001, CI = .03 to .09) and 24 months (*B* = .06, *SE* = .03, *p* = .036, *CI* = .02 to .10). Age and gender did not predict PFS scores or BMI at any other time points.
